# Mediators of the effects of an HIV prevention advocacy training intervention on prevention advocacy among persons living with HIV in Uganda

**DOI:** 10.1007/s10865-026-00704-z

**Published:** 2026-08-31

**Authors:** Glenn J. Wagner, Laura M. Bogart, Joseph K. B. Matovu, Violet Gwokyalya, Harold D. Green, Susan Ninsiima, David J. Klein, Stephen Okoboi

**Affiliations:** 1 RAND Corporation, 1776 Main Street, P.O. Box 2138, Santa Monica, CA 90407-2138 USA; 2 Charles R. Drew University of Medicine and Science, Los Angeles, CA USA; 3 Makerere University School of Public Health, Kampala, Uganda; 4 Busitema University Faculty of Health Sciences, Mbale, Uganda; 5 Indiana University School of Public Health—Bloomington, Bloomington, IN USA; 6 Infectious Diseases Institute, College of Health Sciences, Makerere University, Kampala, Uganda

**Keywords:** HIV, Peer advocacy, Prevention, Internalized HIV stigma, HIV knowledge, Uganda

## Abstract

Pathways through which advocacy training enhances engagement in advocacy among persons living with HIV (PLWH) are not well understood. In a secondary analysis from a randomized controlled trial of a peer advocacy training intervention for PLWH, we examined mediators of intervention effects on HIV prevention advocacy (i.e., encouraging others to engage in HIV protective behaviors) across members of their social network. A sample of 210 PLWH (105 in each of the intervention and control arms) provided information about their advocacy to their social network members at baseline, and 6-, 12-, and 18-month follow-up. Repeated measures linear regressions showed that PLWH in the intervention group targeted more social network members with advocacy for HIV testing, condom use, pre-exposure prophylaxis (PrEP) use, HIV care, and ART use over the follow-up period compared to those in the control group. In path analysis that examined each mediator in a separate model, reduced internalized HIV stigma and increased HIV knowledge as measured at month 6, each fully mediated the intervention effect on HIV care advocacy; reduced stigma partially mediated the effects on advocacy targeting each of the other four HIV protective behaviors, while increased knowledge partially mediated effects on HIV testing, condom and PrEP use advocacy. Results were unchanged when examining the joint mediation of stigma and knowledge. HIV disclosure and advocacy self-efficacy had no mediating effect. These findings highlight the importance of helping PLWH to cope with HIV stigma and gain knowledge about basic facts and common misconceptions related to HIV, to propel their engagement in advocacy for multiple HIV protective behaviors within their social networks.

The trial was registered 2021-10-18 (NCT05098015).

## Introduction

HIV prevalence has remained above 5% in Uganda for over two decades (Uganda AIDS Commission, 2024). About one-quarter of persons living with HIV (PLWH) do not know their HIV status, rates of consistent condom use range from 10 to 40% depending on the population, and access to pre-exposure prophylaxis (PrEP) remains poor (Nabayinda et al., 2023; Nabunya et al., 2023; Ssekamatte et al., 2022; Vithalani & Herreros-Villanueva, 2018). While external HIV stigma has decreased over time (NAFOPHANU, 2019) and HIV disclosure is often met with supportive responses (Atuyambe et al., 2014), HIV stigma nonetheless remains prevalent in Uganda (Jolle et al., 2022; Kuteesa et al., 2014; Namuli et al., 2024). A third to one half of PLWH in Uganda report experiencing some form of HIV-related discrimination or prejudice (Uganda AIDS Commission, 2024; Namuli et al., 2024), such as loss of employment, social insults and ridicule, and relationship abandonment. These challenges to HIV prevention are further exacerbated by the recent drastic reduction in United States government funding for HIV care and prevention in Uganda and other parts of sub-Saharan Africa (UNAIDS, 2025). Innovative, low resource strategies for promoting HIV prevention in Uganda are critical to maintain gains achieved and to make further inroads against HIV.

Peer advocacy for HIV prevention [i.e., discussing HIV with peers and encouraging HIV protective behaviors such as HIV testing or use of condoms, PrEP, and antiretroviral therapy (ART)] is an approach that has been successfully used to promote HIV prevention (Kegeles et al., 1999; Kelly, 2004). Efforts to promote peer advocacy for HIV prevention typically involve recruiting members of a specific at-risk group to encourage other members to reduce risky behaviors and adopt healthy and protective behaviors (Webel, 2010). A recent systematic review and meta-analysis found that peer advocacy was associated with a 36% decrease in HIV infection among high-risk groups, as well as increased HIV testing and condom use, and reduced intravenous needle sharing and unprotected sex (He et al., 2020).

Through peer advocacy for HIV prevention, PLWH have the potential to be change agents within their social networks and represent a “game changer” for HIV prevention, particularly in high-prevalence settings such as Uganda where virtually every family is touched by HIV. Our research suggests that nearly all PLWH engage in some level of prevention advocacy (Ssegujja et al., 2011; Tumwine et al., 2012; Wagner et al., 2024). Unlike mass media campaigns, peer advocacy revolves around an interpersonal bidirectional interaction between peers. PLWH can be especially influential and credible in conveying prevention messages to family and friends, given these close and trusted relations, and their personal lived experience with HIV (Raja et al., 2007).

To maximize the potential impact of peer HIV prevention advocacy performed by PLWH, we developed the Game Changer for HIV Prevention (GC-HIV) advocacy training intervention. GC-HIV is a multi-session group intervention that empowers PLWH to engage in advocacy with people in their social networks. We completed a randomized controlled trial of GC-HIV with 210 PLWH in Uganda (Bogart et al., 2026). The intervention had significant effects on increased engagement in HIV prevention advocacy among enrolled social network members of the PLWH participants, and the network members who received HIV testing and condom use advocacy were more likely to report recent HIV testing and consistent condom use than their counterparts (Wagner et al., 2025). In the analysis reported in this paper, we seek to understand the mechanisms that drive these intervention effects on HIV prevention advocacy.

Game Changers draws on social diffusion theory (Centola, 2018; Valente, 1996) and principles of social influence (Broadhead et al., 1998), which posit that behavior change can be initiated by a few and diffused to others through social norm change. Self-acceptance, through *reduced internalized HIV stigma* (Pantelic et al., 2019), helps facilitate comfort with *disclosing one’s HIV status* and sharing one’s experience of living with HIV (Tsai et al., 2013), which is central to facilitating discussions about HIV prevention (Wagner et al., 2021). *HIV knowledge* is important for accurate advocacy and dispelling myths and misconceptions that are encountered in advocacy conversations. Lastly, learning communication skills and strategies for when, how, and who to engage in advocacy helps to bolster confidence and *self-efficacy for conducting advocacy* (Siril et al., 2019). These constructs are directly targeted by the content of the intervention and are hypothesized to be the mechanisms by which the intervention affects engagement in peer advocacy. Our prior research in Uganda has shown each of these constructs to be associated with engagement in HIV prevention advocacy in cross-sectional analysis (Wagner et al., 2024), and the GC-HIV intervention was found to significantly reduce internalized HIV stigma and increased HIV disclosure, HIV knowledge and advocacy self-efficacy (Bogart et al., 2026).

This paper builds on our prior research by examining effects of the GC-HIV intervention on advocacy performed across participants’ larger social network. We hypothesize that the intervention increases engagement in HIV prevention advocacy with respect to the use of condoms, HIV testing, PrEP, HIV care and ART among the social networks of PLWH, and that each of the four constructs in the intervention’s conceptual model (internalized HIV stigma, HIV disclosure, HIV knowledge, advocacy self-efficacy) would at least partially mediate these effects.

## Methods

### Study design

The study was a parallel randomized controlled trial of the GC-HIV group peer advocacy intervention, conducted at The Infectious Diseases Institute (IDI) in Kampala, Uganda. PLWH were enrolled as index participants and randomized to receive either the intervention or a non-intervention usual care control group using a blocked 1:1 randomization design with stratification by sex. Each index participant was asked to recruit up to four members of their personal social network (henceforth referred to as “alters”) to participate in the study; however, only data from index participants (and not the enrolled alters) were included in the analysis for this paper. After baseline, follow-up assessments were administered at months 6, 12 and 18. Participants received 30,000–70,000 Uganda shillings (~ $8–20 USD), depending on the distance traveled, after completing each assessment, or attending each intervention session, to cover transportation costs. The study protocol was approved by ethical review boards at [RAND (2021-N0155) and IDI (009-2021), and the Uganda National Council for Science and Technology. Participants provided written informed consent. Further details of the study protocol are available in a prior publication (Bogart et al., 2025). The study has been registered with clinicaltrials.gov (NCT05098015, registered 2021-10-18).

### Participants

Recruitment took place between January 2022 and February 2023. Eligibility criteria for index participants included: (1) age ≥18 years, (2) in HIV care for at least one year (so that people have had time to adjust to their HIV diagnosis), (3) speak fluent Luganda or English, and (4) does not have a partner/spouse or household member already enrolled in the study as an index participant.

### Intervention

The intervention consisted of eight weekly group sessions, each of which was attended by 8–10 index participants and lasted about 2 h. The session content focused on use of self-compassion and peer support to overcome internalized stigma, HIV disclosure decision making, HIV facts and myths, healthy positive living with HIV, and learning and practicing skills for HIV prevention advocacy (how to start and sustain conversations about HIV). Prior published papers from the study give greater detail about the content and structure of the intervention sessions (Bogart et al., 2026); here we describe in some detail sessions 5 to 7, which focused on building skills for engaging in HIV prevention advocacy. In these sessions, role playing is used to help participants learn how to start conversations about HIV by looking for opportunities such as “teaching moments” during everyday conversations, or using observations about the environment, and using communication techniques such as open-ended questions, encouragement (rather than discouragement), and rephrasing to both keep conversations going and to work through resistance. Role plays in these latter sessions focused on how to help alters who might benefit from prevention advocacy, regardless of HIV-serostatus (e.g., alters who discussed not using condoms, had multiple partners, showed signs of illness, and expressed belief in misconceptions about HIV). Role plays are prominent in each session as they enable these strategies and techniques to be practiced and observed being performed by others, and facilitators can assess whether skills have been mastered or more practice is needed. Each session also included sharing of experiences to build support; setting personal goals regarding engagement in disclosure and advocacy; and take-home activities to reinforce new skills and generate personal experiences to be processed in the sessions.

The sessions were conducted in Luganda or English (depending on the preference of the participants) by two trained Ugandan peer-facilitators (who were PLWH) using a structured manual. The facilitators were trained by the Ugandan and American senior investigators over three days. To monitor and promote sustained fidelity to the intervention manual, the Ugandan supervisor of the facilitators observed implementation of each session, rated fidelity and completion of each session exercise, and provided feedback and further training as needed during weekly supervision meetings with the two peer-facilitators.

### Measures

Survey and social network assessments were interviewer-administered using Network Canvas software (Birkett et al., 2021) and conducted in Luganda or English, depending on the preference of the participant. All measures were translated from English to Luganda using a standard translation/backtranslation methodology in a pilot study (Bogart et al., 2020). Each assessment administered to index participants included a social network assessment in which the participant was asked to list 20 people in their social network (“alters”) with whom they interact most frequently. For each alter named, the index participant was asked to provide information, as described below. All measures were administered at each assessment, unless otherwise noted.

**Index participant engagement in HIV prevention advocacy.** For each alter named in the social network assessment, index participants were asked whether they engaged in discussions with the alter about the use of specific behaviors that promote primary (prevent viral acquisition) or secondary (prevent transmission of infection) prevention of HIV. In separate yes/no questions using the preamble “Have you talked with [alter name] about …”, index participants were asked if they had talked with the alter in the past three months about (1) condom use, (2) getting tested for HIV or (3) use of PrEP if the alter was not living with HIV, and if the alter was living with HIV—(4) engaging in HIV care or (5) taking ART. For each of these five types of advocacy, the percentage of alters targeted with advocacy was calculated at each assessment; the average percentage across all three follow-up assessments was calculated to represent the primary dependent variable for analyses.

**Internalized HIV stigma** was measured using the 8-item Internalized AIDS-Related Stigma Scale (Kalichman et al., 2009). Examples of items include "Being HIV-positive makes me feel like something is wrong with me" and "I feel guilty that I am HIV-positive". Response options range from 1 ‘disagree strongly’ to 5 ‘agree strongly’. The mean item score was calculated and higher scores represent greater stigma (Cronbach’s alpha = .80).

**HIV disclosure** was measured by calculating the percentage of alters to whom the participant reported having disclosed their HIV status.

**HIV knowledge** was assessed with 13 statements related to the goals of HIV medication (e.g., “If a person with HIV infection does not take medication for HIV, their HIV viral load will increase”), drug resistance (e.g., “If you do not take HIV medication exactly as instructed, HIV in your body may become resistant to HIV medications”), adherence (e.g., “An HIV-positive individual does not need to take medication for HIV everyday if they do not have any symptoms”), HIV myths and misconceptions (e.g., “A person can get HIV through witchcraft of other supernatural means”), and HIV prevention (e.g., “Having an undetectable HIV viral load makes it very difficult for you to transmit the virus to someone else”). Some items were developed by the study team, while others were derived from the Patient's HIV Knowledge Questionnaire (Jackson et al., 2020). Response options consisted of ‘true,’ ‘false,’ ‘don't know,’ and ‘not sure,’ and a sum of correct responses was calculated.

**Self-efficacy for HIV prevention advocacy** was measured with a single item in which participants rated their confidence in being able to start a conversation about HIV with people they know on a scale of 0 ‘cannot do at all’ to 10 ‘certain I can do’.

### Background characteristics

Index participants reported their age (in years), sex (male, female), highest level of education, relationship status, and length of time since HIV diagnosis. For each alter named in their social network assessment, index participants reported age, sex, nature of relationship with the alter [i.e., family, friend, other (e.g., coworker)], and perceived HIV status.

### Data analysis

Bivariate statistics (independent 2-tailed t-tests; chi-square tests) were used to compare background characteristics of index participants and named alters between those in the intervention and controls groups. To test intervention effects on the primary dependent variables (average percent of alters targeted with the specific type of advocacy across the three follow-up assessments), we compared these measures between the intervention and control arms using linear regression analysis and controlling for index age, sex, secondary education, relationship status, and the baseline measure of the dependent variable. Separate models were conducted for each of the five types of advocacy. We used the same approach to examine intervention effects on the four potential mediators [internalized HIV stigma, HIV disclosure, HIV knowledge, and HIV prevention advocacy self-efficacy], with separate models for each mediator and the mediator measured at month 6 as the dependent variable.

If the regression models described above revealed a significant intervention effect on the type of advocacy, analysis was performed to assess whether the effect was mediated by any of the potential mediators. For each primary dependent variable (average percentage of named alters who were targeted with the specific type of advocacy across the three follow-up assessments) and potential mediator, a path analysis was conducted, with three functional relationships specified. The first path specified that the intervention, expressed as an indicator of study arm, affected the primary dependent variable as well as the mediator as measured at month 6. The second path specified that the mediator as measured at month 6 affected the primary dependent variable. The third path specified that index characteristics (age, secondary education, sex, and relationship status), the mediator as measured at baseline, and the baseline measure of the dependent variable affected the primary dependent variable as well as the mediator as measured at month 6. The index sociodemographic characteristics were included as covariates so that the findings could be generalizable to PLWH regardless of these characteristics. Regression coefficients for all paths as well as for direct and indirect effects were estimated with SAS v9.4 Proc Calis. These models were run for each mediator individually, in separate analyses. However, if multiple mediators were found to partially mediate the intervention effect on the primary dependent variable, a subsequent model was run that included those mediators simultaneously (measured at baseline and month 6) to assess whether jointly these mediators would fully mediate the intervention effect on advocacy. Given that the mediators were variables measured at month 6, the overall analytic sample was restricted to index participants who completed the month 6 assessment. Two index participants did not complete the month 6 assessment and were excluded from the analysis. Lastly, with four mediators examined for each of five dependent variables, we acknowledge the potential for Type 1 errors; however, we justify not adjusting for multiple comparisons because we consider these models to be confirmatory in nature as they examine relationships that correspond to our conceptual framework.

## Results

### Sample characteristics

Two hundred and ten index participants (105 in each of the intervention and control arms) enrolled in the study, of whom 208 completed the month 6 assessment and comprised the analytic sample. These 208 index participants named 3,185 alters at baseline (mean = 15.3 alters per index participant in intervention arm vs. 15.4 in control arm; *p* = .91). Table 1 shows background characteristics of index participants and their named alters, in the whole sample as well as within the control and intervention arms; there were no statistically significant differences between arms on any of these characteristics. Among index participants, mean age was near 40 years, roughly a third were male, the majority had at least some secondary education and two-thirds were married or in a committed relationship. Among alters, about one-fifth were living with HIV and the index had disclosed their HIV status to half; roughly half were family members, while most of the remaining were friends.

**Table 1:** Baseline characteristics of index participants and their named social network members in the whole sample and within each study arm

	Total	Control	Intervention	*p*
*Characteristics of index participants*				
Number of index participants	208	103	105	
Age	40.0 (10.6)	39.3 (10.5)	40.7 (10.7)	.36
Male	63 (30.3%)	32 (31.1%)	31 (29.5%)	.81
Any secondary education	124 (59.6%)	67 (65.1%)	57 (54.3%)	.11
Married or in committed relationship	138 (66.4%)	68 (66.0%)	70 (66.7%)	.92
Years since HIV diagnosis				
Internalized HIV stigma	2.3 (0.9)	2.3 (0.9)	2.4 (1.0)	.57
HIV knowledge	10.6 (1.4)	10.5 (1.3)	10.7 (1.4)	.20
HIV prevention advocacy self-efficacy	7.1 (2.2)	7.3 (2.1)	7.0 (2.2)	.38
*Characteristics of alters*^a^				
Number of alters per index	15.3 (4.9)	15.4 (4.8)	15.3 (5.0)	.91
Mean age	37.5 (5.7)	38.0 (6.0)	37.0 (5.4)	.17
% male	42.7 (18.9)	45.2 (19.2)	40.3 (18.3)	.06
% perceived to be living with HIV	19.5 (16.1)	20.5 (16.7)	18.5 (15.4)	.35
% disclosed HIV status to by index (HIV disclosure)	51.2 (32.2)	48.5 (31.1)	53.9 (33.1)	.23
% family of index	47.4 (22.3)	47.7 (23.2)	47.1 (21.6)	.85
% friends of index	51.7 (22.2)	51.9 (23.3)	51.5 (21.3)	.90

The *p* values are based on t-test for continuous characteristics and chi-square tests for binary characteristics

^a^The characteristics of alters are represented by data at the level of the index participant; each characteristic represents an average across all alters of an index, and then the average of that value across all index participants

### Intervention effects on HIV prevention advocacy

Controlling for advocacy reported at baseline and index background characteristics (age, secondary education, sex, and relationship status), index participants in the intervention group reported targeting a greater percentage of alters during the follow-up period for each of the five forms of advocacy, compared to those in the control group: condom use [32.4% vs. 20.7%;

beta (SE) = 11.78 (2.63); *p* < .001], HIV testing [40.1% vs. 26.5%; beta (SE) = 13.72 (2.99); *p* < .001], PrEP use [20.3% vs. 7.7%; beta (SE) = 12.52 (2.27); *p* < .001], HIV care engagement [51.7% vs. 43.1%; beta (SE) = 11.20 (4.13); *p* = .007] and ART use [62.3% vs. 51.3%; beta (SE) = 12.62 (3.93); *p* = .002].

### Mediation of the intervention effects on HIV prevention advocacy

Controlling for the baseline measure of the mediator and index background characteristics, the intervention had a significant effect on each of the four potential mediators at month 6: internalized HIV stigma [beta (SE) = − 0.50 (0.09); *p* < .001], HIV disclosure [beta (SE) = 6.86 (2.95); *p* = .02], HIV knowledge [beta (SE) = 0.47 (0.16); *p* = .004], and HIV prevention advocacy self-efficacy [beta (SE) = 1.16 (0.30); *p* < .001], with separate models for each mediator.

Tables 2, 3, 4, 5 and 6 present the results of the path analysis examining mediation across the five types of HIV prevention advocacy, with each mediator examined separately. These analyses revealed that reduced internalized HIV stigma was significantly associated with increased advocacy with respect to each of the five HIV protective behaviors. Furthermore, internalized HIV stigma fully mediated the intervention effect on HIV care advocacy, with a significant indirect effect [beta (SE) = 4.24 (1.71); *p* = .01] and nonsignificant direct effect [beta (SE) = 7.06 (4.22); *p* = .09] (see Table 4). For each of the other four types of advocacy, internalized HIV stigma partially mediated the intervention effect, with both a significant indirect effect and significant direct effect.

**Table 2:** Regression models examining change (from baseline to month 6) in internalized HIV stigma, HIV disclosure, HIV knowledge, and HIV prevention advocacy self-efficacy as mediators of the intervention effect on engagement in HIV testing advocacy among HIV-negative social network members across all 18 months of follow-up (n = 208)

	Internalized HIV Stigma		HIV Disclosure		HIV Knowledge		HIV Prevention Advocacy Self-Efficacy		Combined model	
	Beta (SE)	*p*	Beta (SE)	*p*	Beta (SE)	*p*	Beta (SE)	*p*	Beta (SE)	*p*
Intervention (Direct effect)	9.79 (3.03)	.001	12.17 (2.91)	< .001	11.56 (2.96)	< .001	12.81 (2.99)	< .001	8.64 (3.07)	.005
% alters targeted with HIV testing advocacy (BL)	0.14 (0.06)	.02	0.12 (0.06)	.047	0.10 (0.06)	.11	0.10 (0.06)	.12	0.12 (0.06)	.06
Internalized HIV stigma (BL)	0.13 (1.67)	.94	–		–		–		0.15 (1.66)	.93
Internalized HIV stigma (M6)	− 8.30 (2.16)	< .001	–		–		–		− 7.26 (2.14)	< .001
HIV disclosure (BL)	–		− 0.12 (0.06)	.049	–		–		–	
HIV disclosure (M6)	–		0.23 (0.07)	.001	–		–		–	
HIV knowledge (BL)	–		–		− 0.05 (1.17)	.96	–		0.30 (1.14)	.80
HIV knowledge (M6)	–		–		3.84 (1.28)	.003	–		2.73 (1.25)	.03
HIV prevention advocacy self-efficacy (BL)	–		–		–		1.48 (0.69)	.03	–	
HIV prevention advocacy self-efficacy (M6)	–		–		–		1.11 (0.69)	.10	–	
Indirect effect	4.13 (1.31)	.002	1.56 (0.81)	.054	1.83 (0.86)	.03	1.30 (0.86)	.13	4.94 (1.46)	< .001

All models assess the indirect effect of the intervention through the mediator(s) at month 6, controlling for the baseline measure of the mediator and the following covariates: age, secondary education, sex and relationship status of index participant

**Table 3:** Regression models examining change (from baseline to month 6) in internalized HIV stigma, HIV disclosure, HIV knowledge, and HIV prevention advocacy self-efficacy as mediators of the intervention effect on engagement in condom use advocacy among social network members across 18 months of follow-up (n=208)

	Internalized HIV Stigma		HIV Disclosure		HIV Knowledge		HIV Prevention Advocacy Self-Efficacy	
	Beta (SE)	*p*	Beta (SE)	*p*	Beta (SE)	*p*	Beta (SE)	*p*
Intervention (Direct effect)	8.04 (2.65)	.002	10.75 (2.57)	< .001	10.42 (2.61)	< .001	11.63 (2.64)	< .0001
% alters targeted with condom use advocacy (BL)	0.30 (0.06)	< .001	0.30 (0.06)	< .001	0.29 (0.06)	< .001	0.27 (0.06)	< .0001
Internalized HIV stigma (BL)	0.31 (1.46)	.83	–		–		–	
Internalized HIV stigma (M6)	− 7.82 (1.89)	< .001	–		–		–	
HIV disclosure (BL)	–		− 0.12 (0.05)	.03	–		–	
HIV disclosure (M6)	–		0.17 (0.06)	.006	–		–	
HIV knowledge (BL)	–		–		− 0.80 (1.03)	.44	–	
HIV knowledge (M6)	–		–		2.88 (1.13)	.01	–	
HIV prevention advocacy self-efficacy (BL)	–		–		–		1.43 (0.61)	.02
HIV prevention advocacy self-efficacy (M6)	–		–		–		0.46 (0.61)	.45
Indirect effect	3.92 (1.18)	< .001	1.14 (0.64)	.07	1.36 (0.70)	.052	0.53 (0.72)	.46

All models assess the indirect effect of the intervention through the mediator at month 6, controlling for the baseline measure of the mediator and the following covariates: age, secondary education, sex and relationship status of index participant

**Table 4:** Regression models examining change (from baseline to month 6) in internalized HIV stigma, HIV disclosure, HIV knowledge, and HIV prevention advocacy self-efficacy as mediators of the intervention effect on engagement in HIV care advocacy among social network members living with HIV across 18 months of follow-up (n = 180)

	Internalized HIV Stigma		HIV Disclosure		HIV Knowledge		HIV Prevention Advocacy Self-Efficacy		Combined model	
	Beta (SE)	*p*	Beta (SE)	*p*	Beta (SE)	*p*	Beta (SE)	*p*	Beta (SE)	*p*
Intervention (Direct effect)	7.06 (4.22)	.09	9.89 (4.13)	.02	7.45 (4.02)	.06	10.77 (4.25)	.01	4.22 (4.21)	.32
% alters targeted with HIV care engagement advocacy (BL)	0.13 (0.05)	.01	0.09 (0.05)	.06	0.09 (0.05)	.051	0.10 (0.05)	.06	0.12 (0.05)	.01
Internalized HIV stigma (BL)	− 0.10 (2.38)	.97	–		–		–		− 0.21 (2.30)	.93
Internalized HIV stigma (M6)	− 8.71 (3.07)	.005	–		–		–		− 8.01 (2.96)	.007
HIV disclosure (BL)	–		− 0.04 (0.09)	.67	–		–		–	
HIV disclosure (M6)	–		0.13 (0.10)	.17	–		–		–	
HIV knowledge (BL)	–		–		3.29 (1.51)	.03	–		3.85 (1.49)	.01
HIV knowledge (M6)	–		–		4.56 (1.77)	.01	–		3.38 (1.74)	.052
HIV prevention advocacy self-efficacy (BL)	–		–		–		0.84 (1.05)	.42	–	
HIV prevention advocacy self-efficacy (M6)	–		–		–		0.73 (0.98)	.46	–	
Indirect effect	4.24 (1.71)	.01	1.20 (0.97)	.21	2.51 (1.23)	.04	0.93 (1.27)	.46	5.83 (1.99)	.003

All models assess the indirect effect of the intervention through the mediator(s) at month 6, controlling for the baseline measure of the mediator and the following covariates: age, secondary education, sex and relationship status of index participant

**Table 5:** Regression models examining change (from baseline to month 6) in internalized HIV stigma, HIV disclosure, HIV knowledge, and HIV prevention advocacy self-efficacy as mediators of the intervention effect on engagement in ART use advocacy among social network members living with HIV across 18 months of follow-up (n = 180)

	Internalized HIV Stigma		HIV Disclosure		HIV Knowledge		HIV Prevention Advocacy Self-Efficacy	
	Beta (SE)	*p*	Beta (SE)	*p*	Beta (SE)	*p*	Beta (SE)	*p*
Intervention (Direct effect)	9.05 (3.99)	.02	10.47 (3.88)	.007	10.62 (3.95)	.007	10.40 (4.01)	.01
% alters targeted with ART use advocacy (BL)	0.18 (0.05)	< .001	0.17 (0.05)	< .001	0.16 (0.05)	< .001	0.15 (0.05)	< .001
Internalized HIV stigma (BL)	− 2.40 (2.25)	.29	–		–		–	
Internalized HIV stigma (M6)	− 7.68 (2.87)	.008	–		–		–	
HIV disclosure (BL)	–		− 0.07 (0.08)	.38	–		–	
HIV disclosure (M6)	–		0.23 (0.09)	.02	–		–	
HIV knowledge (BL)	–		–		− 1.09 (1.49)	.46	–	
HIV knowledge (M6)	–		–		3.70 (1.74)	.03	–	
HIV prevention advocacy self-efficacy (BL)	–		–		–		− 0.01 (0.99)	.99
HIV prevention advocacy self-efficacy (M6)	–		–		–		1.96 (0.93)	.04
Indirect effect	3.73 (1.58)	.02	1.99 (1.07)	.06	2.04 (1.13)	.07	2.49 (1.33)	.06

All models assess the indirect effect of the intervention through the mediator at month 6, controlling for the baseline measure of the mediator and the following covariates: age, secondary education, sex and relationship status of index participant

**Table 6:** Regression models examining change (from baseline to month 6) in internalized HIV stigma, HIV disclosure, HIV knowledge, and HIV prevention advocacy self-efficacy as mediators of the intervention effect on engagement in PrEP use advocacy among HIV-negative social network members across 18 months of follow-up (n = 208)

	Internalized HIV Stigma		HIV Disclosure		HIV Knowledge		HIV Prevention Advocacy Self-Efficacy		Combined model	
	Beta (SE)	*p*	Beta (SE)	*p*	Beta (SE)	*p*	Beta (SE)	*p*	Beta (SE)	*p*
Intervention (Direct effect)	10.68 (2.31)	< .001	11.64 (2.23)	< .001	10.49 (2.21)	< .001	11.98 (2.22)	< .001	9.44 (2.31)	< .001
% alters targeted with PrEP use advocacy (BL)	0.42 (0.09)	< .001	0.45 (0.09)	< .001	0.42 (0.09)	< .001	0.39 (0.09)	< .001	0.40 (0.09)	< .001
Internalized HIV stigma (BL)	− 2.29 (1.27)	.07	–		–		–		− 2.24 (1.25)	.07
Internalized HIV stigma (M6)	− 4.19 (1.64)	.01	–		–		–		− 3.19 (1.60)	.047
HIV disclosure (BL)	–		0.01 (0.05)	.90	–		–		–	
HIV disclosure (M6)	–		0.11 (0.05)	.04	–		–		–	
HIV knowledge (BL)	–		–		0.25 (0.86)	.77	–		0.49 (0.84)	.56
HIV knowledge (M6)	–		–		3.49 (0.96)	< .001	–		2.81 (0.94)	.003
HIV prevention advocacy self-efficacy (BL)	–		–		–		1.75 (0.52)	< .001	–	
HIV prevention advocacy self-efficacy (M6)	–		–		–		0.81 (0.51)	.11	–	
Indirect effect	2.11 (0.91)	.02	0.72 (0.47)	.12	1.67 (0.71)	.02	0.93 (0.63)	.14	2.98 (1.07)	.005

All models assess the indirect effect of the intervention through the mediator(s) at month 6, controlling for the baseline measure of the mediator and the following covariates: age, secondary education, sex and relationship status of index participant

Similarly, increased HIV knowledge was significantly associated with increased advocacy for each of the five HIV protective behaviors. Like stigma, HIV knowledge fully mediated the intervention effect on HIV care advocacy, with a significant indirect effect [beta (SE) = 2.51 (1.23); *p* = .04] and nonsignificant direct effect [beta (SE) = 7.45 (4.02); *p* = .06] (see Table 4). It partially mediated the effect of the intervention on HIV testing and PrEP use advocacy, with both a significant indirect effect and significant direct effect, but it played no mediating role in the effects on condom and ART use advocacy.

Increased HIV disclosure was significantly associated with increased advocacy with respect to condom use, HIV testing, PrEP use, and ART use, but not HIV care advocacy. However, HIV disclosure did not mediate the intervention effect on any of these forms of advocacy, as the indirect effect was nonsignificant in each model. Increased self-efficacy for HIV prevention advocacy was significantly associated with increased ART use advocacy only (see Table 5), and it did not act as a mediator of the intervention effect on any of the types of advocacy.

With the above results showing that reduced stigma and increased knowledge had each at least partially mediated the intervention effects on three forms of advocacy (HIV testing, HIV care and PrEP), both mediators were then included in the analysis to assess their roles as joint mediators. With both mediators in the model, the intervention effect on increased HIV care advocacy was fully mediated (see Table 4), with both a significant indirect effect and insignificant direct effect; however, the effects on HIV testing (see Table 2) and PrEP use (see Table 6) advocacy remained only partially mediated, with both the indirect and direct effects being significant. The results of each of these combined models with the joint mediators are depicted in separate diagrams in the online Supplementary Materials.

## Discussion

In this secondary analysis of data from a randomized controlled trial of the GC-HIV intervention, results showed that the intervention had a significant effect on increased advocacy for each of the five HIV protectives behaviors examined. Each of the four components of the intervention that were hypothesized to mediate the effects on advocacy (i.e., internalized HIV stigma, HIV disclosure, HIV knowledge, advocacy self-efficacy) was associated with increased engagement in at least one (and in most cases, several) form of HIV prevention advocacy. Reduced internalized HIV stigma and increased HIV knowledge were each found to be significantly associated with increased advocacy for all five of the HIV protective behaviors; furthermore, both were found to fully mediate the intervention effects on increased HIV care advocacy, as well as partially mediate several of the other forms of HIV prevention advocacy, highlighting their particular importance as a focus of the advocacy training intervention.

Prior research has shown that nearly all PLWH can be expected to engage in some level of HIV prevention advocacy with people they care about (Tumwine et al., 2012; Wagner et al., 2024). This is further supported by our data; PLWH in the control group reported HIV prevention advocacy with many of their alters within the three months prior to baseline and across the follow-up assessments. However, those in the intervention arm reported advocacy targeting each of the five examined HIV protective behaviors (condom use, HIV testing, PrEP use, HIV care engagement, ART use) with a significantly higher percentage of their alters at follow-up. Advocacy for HIV care and ART use among alters who were also living with HIV were particularly high (50–60% of alters), while slightly lower proportions of alters (30–40%) were targeted with condom use and HIV testing advocacy. Although PrEP is not widely available in Uganda, it is noteworthy that one-fifth of alters not living with HIV were targeted with PrEP use advocacy in the intervention group. The greater prevalence of advocacy for behaviors that were relevant for alters living with HIV may be attributed to PLWH being more comfortable discussing HIV with and disclosing to peers who are also PLWH. These findings, which account for advocacy conducted across the larger social network of each participating PLWH, are consistent with and expand upon our prior findings from the trial that showed intervention effects on these types of advocacy among the few alters who were enrolled in the study (Wagner et al., 2025).

Consistent with the conceptual framework of the intervention, and our prior published findings that the intervention had its designed effects on reduced internalized HIV stigma and increased HIV disclosure, HIV knowledge and advocacy self-efficacy (Bogart et al., 2026), these effects were also observed at the month 6 assessment—the time frame for which these effects were hypothesized to mediate the intervention effects on increased HIV prevention advocacy. Change in each of these four constructs was significantly associated with increased advocacy for at least one of the five examined HIV protective behaviors, and increased advocacy for all five protective behaviors was associated with both reduced internalized stigma and increased HIV knowledge. Change in stigma and knowledge were each found to fully mediate the intervention effects on HIV care advocacy. Further, reduced stigma partially mediated the intervention effects on each of the other four types of advocacy, while increased knowledge partially mediated effects on HIV testing and PrEP use advocacy. Reduced internalized HIV stigma and increased HIV knowledge may be particularly important for promoting advocacy engagement because the former is central to PLWH being comfortable initiating discussions about how to reduce HIV risk and sharing their personal lived HIV experience, while the latter enables a PLWH to feel confident that they can offer accurate information and guidance to their peers about how to protect themselves against HIV.

Increased HIV disclosure and advocacy self-efficacy had no mediating role on the advocacy effects of the intervention. While change in HIV disclosure and self-efficacy by month 6 were each significantly associated with an increase in various types of advocacy, a more distal measure of change in these constructs may be needed to reveal a mediating role. Furthermore, we conceptualize comfort with HIV disclosure as being both predicated on low enough internalized HIV stigma and being a precursor to engaging in HIV prevention advocacy and sharing personal HIV-related experience; therefore, the process of HIV disclosure may overlap with these processes and not be an adequately distinct mechanism for a mediating role to emerge. Sharing personal lived experiences with HIV is a component of both HIV disclosure and HIV prevention advocacy. It is plausible to conceive HIV disclosure as an actual form of HIV prevention advocacy and an initial stage of advocacy that leads to a subsequent cascade of advocacy behaviors (e.g., encouraging others to engage in HIV protective behaviors), rather than a mechanism that drives subsequent advocacy engagement.

These findings have implications for theory, design and implementation of the intervention. The findings suggest that internalized HIV stigma reduction and increased HIV knowledge may have greater importance in promoting HIV prevention advocacy among PLWH compared to the other components of the intervention (e.g., HIV disclosure decision making); therefore, future iterations of this advocacy training model could consider proportionately increasing the resources devoted to these components. Future studies of the intervention could explore whether implementing the intervention with PLWH who have higher internalized stigma may result in even greater gains in advocacy given the intervention’s demonstrated ability to reduce stigma; however, it is possible that PLWH with higher stigma would be less interested in joining such a group, participate less in the sessions, and receive less benefit with regards to stigma reduction and advocacy engagement compared to those with less stigma. The findings also contribute to theories of social diffusion and social influence by providing empirical support for the influence of these constructs on multiple forms of HIV prevention advocacy engagement– particularly the roles of reduced internalized HIV stigma and increased HIV knowledge as psychological mechanisms through which PLWH can engage in more HIV prevention peer advocacy to affect behavioral change. However, the constructs in our conceptual framework do not fully explain how the intervention impacts engagement in wide array of types of advocacy. Other unmeasured factors may be at play, such as aspects of the relationship between the index and alter, highlighting the need for further research to improve our understanding of the context of prevention advocacy discussions.

Other study limitations should be noted. The primary dependent variable, engagement in HIV prevention advocacy, relied solely on the subjective self-report of the index participants not objective measures (e.g., alter verified discussions or observational methods), which could be biased by social desirability, particularly among index participants in the intervention arm. The advocacy measure only assessed the presence of a discussion about the alter use of a specific HIV protective behavior, not other aspects such as the duration of the discussion and how many discussions took place, or qualities of the communication between the index and alter during the discussion. While these measurement shortcomings highlight how future research could further develop this line of work, it is noteworthy that the simple measure used revealed many significant associations among the studied constructs. As alluded to above, added measures of the mediators at more distal time points from baseline would better enable the study to examine whether mediation effects are sustained, diminish or emerge over longer follow-up periods. The generalizability of our findings is limited by the selection bias related to index participants needing to be in HIV care at an HIV research center of excellence for at least the past year, and the inherent motivation of such participants to participate in advocacy training and research—which likely differ from PLWH not in care or the general population of PLWH. Also, the measure of advocacy self-efficacy consisted of only a single item that assessed general engagement in HIV-related discussions. A measure that assessed self-efficacy regarding a wider array of communication skills (e.g., nonjudgmental tone) that pertain to engagement in advocacy, or that was more specific to the target HIV protective behavior (e.g., confidence in talking to others about PrEP), may be needed to better assess the role of this construct.

In conclusion, these findings demonstrate the effectiveness of the GC-HIV peer advocacy training intervention for empowering PLWH to engage in more HIV prevention advocacy within their social networks, promoting HIV protective behaviors that are relevant for sexually active social network members (condom use), those who are not living with HIV (HIV testing, PrEP use), and those living with HIV (engagement in HIV care, ART use). Given the high HIV prevalence in Uganda, advocacy for HIV prevention by PLWH has the potential for wide dissemination of HIV prevention messaging and information across networks and communities. This positions PLWH to play a key role in combatting HIV. The intervention was found to increase engagement in HIV prevention advocacy, in part through its effects on reduced internalized HIV stigma and increased HIV knowledge, which either fully or partially mediated the intervention’s effect depending on the type of advocacy. These findings highlight the importance of helping PLWH to cope with HIV stigma and gain knowledge about basic facts and common misconceptions related to HIV, which can propel their engagement in advocacy for HIV prevention behaviors within their social networks.

## Data Availability

A de-identified dataset will be made available to researchers upon review of written request.

## References

[CR1] Atuyambe, L. M., Ssegujja, E., Ssali, S., Tumwine, C., Nekesa, N., Nannungi, A., et al. (2014). HIV/AIDS status disclosure increases support, behavioural change and, HIV prevention in the long term: A case for an urban clinic, Kampala, Uganda. *BMC Health Services Research,**14*, Article 276. 10.1186/1472-6963-14-276 PMC4076501 24950958

[CR2] Birkett, M., Melville, J., Janulis, P., Phillips, G., Contractor, N., & Hogan, B. (2021). Network canvas: Key decisions in the design of an interviewer-assisted network data collection software suite. *Social Networks,**66*, 114–124. 10.1016/j.socnet.2021.02.003 PMC8153363 34054204

[CR3] Bogart, L. M., Matovu, J. K. B., Green, H. D., Jr., Ninsiima, S., Klein, D. J., Gwokyalya, V., et al. (2026). Randomized controlled trial of Game Changers, a social network intervention for HIV prevention in Uganda. *AIDS and Behavior,**30*(4), 1007–1021. 10.1007/s10461-025-04907-4 PMC12614433 41214256

[CR4] Bogart, L. M., Matovu, J. K. B., Wagner, G. J., Green, H. D., Storholm, E. D., Klein, D. J., Marsh, T., Maccarthy, S., & Kambugu, A. (2020). A pilot test of Game Changers, a social network intervention to empower people with HIV to be prevention advocates in Uganda. *AIDS and Behavior,**24*(9), 2490–2508. 10.1007/s10461-020-02806-4 PMC7415589 32030525

[CR5] Broadhead, R. S., Heckathorn, D. D., Weakliem, D. L., Anthony, D. L., Madray, H., Mills, R. J., & Hughes, J. (1998). Harnessing peer networks as an instrument for AIDS prevention: Results from a peer-driven intervention. *Public Health Reports,**113*(Suppl 1), 42–57. PMC1307726 9722809

[CR6] Centola, D. (2018). *How behavior spreads: The science of complex contagions*. Princeton University Press.

[CR7] Green, H. D., Jr., Hoover, M. A., Wagner, G. J., Ryan, G. W., & Ssegujja, E. (2014). Measuring agreement between egos and alters: Understanding informant accuracy in personal network studies. *Field Methods,**26*(2), 126–140. 10.1177/1525822x13492676 PMC11415257 39310852

[CR8] He, J., Wang, Y., Du, Z., Liao, J., He, N., & Hao, Y. (2020). Peer education for HIV prevention among high-risk groups: A systematic review and meta-analysis. *BMC Infectious Diseases,**20*(1), 338. 10.1186/s12879-020-05003-9 PMC7218508 32398032

[CR9] Jackson, I. L., Okonta, J. M., & Ukwe, C. V. (2020). Development and psychometric evaluation of the patient’s HIV knowledge questionnaire (PHKQ). *International Journal of Clinical Pharmacy,**42*(2), 695–702. 10.1007/s11096-020-00963-z 31939032

[CR10] Jolle, J., Kabunga, A., Okello, T. O., Kadito, E. O., Aloka, J., Otiti, G., et al. (2022). HIV-related stigma experiences and coping strategies among pregnant women in rural Uganda: A qualitative descriptive study. *PLoS ONE,**17*(10), Article e0272931. 10.1371/journal.pone.0272931 PMC9543605 36206276

[CR11] Kalichman, S. C., Simbayi, L. C., Cloete, A., Mthembu, P. P., Mkhonta, R. N., & Ginindza, T. (2009). Measuring AIDS stigmas in people living with HIV/AIDS: The Internalized AIDS-Related Stigma Scale. *AIDS Care,**21*(1), 87–93. 10.1080/09540120802032627 19085224

[CR12] Kegeles, S. M., Hays, R. B., Pollack, L. M., & Coates, T. J. (1999). Mobilizing young gay and bisexual men for HIV prevention: A two-community study. *AIDS,**13*(13), 1753–1762. 10.1097/00002030-199909100-00020 10509578

[CR13] Kelly, J. A. (2004). Popular opinion leaders and HIV prevention peer education: Resolving discrepant findings, and implications for the development of effective community programmes. *AIDS Care,**16*(2), 139–150. 10.1080/09540120410001640986 14676020

[CR14] Kuteesa, M. O., Wright, S., Seeley, J., Mugisha, J., Kinyanda, E., Kakembo, F., et al. (2014). Experiences of HIV-related stigma among HIV-positive older persons in Uganda--a mixed methods analysis. *SAHARA Journal,**11*(1), 126–137. 10.1080/17290376.2014.938103 PMC4272102 25053275

[CR15] Nabayinda, J., Kizito, S., Witte, S., Nabunnya, P., Kiyingi, J., Namuwonge, F., et al. (2023). Factors associated with consistent condom use among women engaged in sex work: Lessons from the Kyaterekera Study in southwestern Uganda. *AIDS and Behavior,**27*(3), 969–977. 10.1007/s10461-022-03833-z PMC9974574 36112259

[CR16] Nabunya, R., Karis, V. M. S., Nakanwagi, L. J., Mukisa, P., & Patience, A. (2023). Barriers and facilitators to oral PrEP uptake among high-risk men after HIV testing at workplaces in Uganda: A qualitative study. *BMC Public Health,**23*, 365. 10.1186/s12889-023-15260-3 PMC9940677 36805698

[CR17] NAFOPHANU. (2019). The PLHIV Stigma Index, Country Assessment, Uganda.

[CR18] Namuli, J. D., Nakasujja, N., Kiyimba, B., Ssewamala, F., McKay, M., Nakimuli-Mpungu, E., & Nakigudde, J. (2024). The burden of HIV stigma and associated factors among HIV infected adolescents attending HIV clinics in Kampala City: A cross-sectional study in Uganda. *EC Psychol Psychiatr,**13*, 01133. PMC12742984 41458631

[CR19] Pantelic, M., Sprague, L., & Stangl, A. L. (2019). It’s not “all in your head”: Critical knowledge gaps on internalized HIV stigma and a call for integrating social and structural conceptualizations. *BMC Infectious Diseases*. 10.1186/s12879-019-3704-1 PMC6399894 30832613

[CR20] Raja, S., McKirnan, D., & Glick, N. (2007). The treatment advocacy program-Sinai: A peer-based HIV prevention intervention for working with African American HIV-infected persons. *AIDS and Behavior,**11*(5), S127–S137. 10.1007/s10461-007-9226-7 17436076

[CR21] Siril, H., Kaale, A., Minja, A., Kilewo, J., Mugusi, F., Sunguya, B., Todd, J., Kaaya, S., & Smith Fawzi, M. C. (2019). Improving communication about HIV prevention among people living with HIV and their at-risk social network members in Dar es Salaam, Tanzania. *Cogent Medicine,**6*(1), Article 1600230. 10.1080/2331205x.2019.1600230 PMC6660010 31355302

[CR22] Ssekamatte, T., Mugambe, R. K., Nalugya, A., Isunju, J. B., Matovu, J. K. B., Kansiime, W. K., et al. (2022). Predictors of consistent condom use among young psychoactive substance users in Kampala’s informal settlements. *Uganda. Dialogues Health,**1*, Article 100080. 10.1016/j.dialog.2022.100080 PMC10953897 38515898

[CR23] Tsai, A. C., Bangsberg, D. R., Kegeles, S. M., Katz, I. T., Haberer, J. E., Muzoora, C., Kumbakumba, E., Hunt, P. W., Martin, J. N., & Weiser, S. D. (2013). Internalized stigma, social distance, and disclosure of HIV seropositivity in rural Uganda. *Annals of Behavioral Medicine,**46*(3), 285–294. 10.1007/s12160-013-9514-6 PMC3795853 23690283

[CR24] Tumwine, C., Nannungi, A., Ssegujja, E., Nekesa, N., Ssali, S., Atuyambe, L., Ryan, G., & Wagner, G. J. (2011). An exploratory study of HIV-prevention advocacy by persons in HIV care in Uganda. *African Journal of AIDS Research,**10*(4), 427–433. 10.2989/16085906.2011.646658 PMC4047788 24910590

[CR25] Tumwine, C., Tumwesigye, N., Mugyenyi, P., & Wagner, G. J. (2012). Psychosocial characterization of HIV clients with potential to be change agents for HIV prevention in Uganda. *Journal of Clinical Research in HIV and AIDS Prevention,**1*(1), 3–11. 10.14302/issn.2324-7339.jcrhap-12-28

[CR26] Uganda AIDS Commission. (2024). *Uganda HIV & AIDS Factsheet*.

[CR27] UNAIDS. (2025). *Impact of US funding cuts on HIV programmes in East and Southern Africa.* March 31, 2025.

[CR28] Valente, T. W. (1996). Network models of the diffusion of innovations. *Computers in Mathematics and Organization Theory,**2*(2), 163–164. 10.1007/BF00240425

[CR29] Vithalani, J., & Herreros-Villanueva, M. (2018). HIV epidemiology in Uganda: Survey based on age, gender, number of sexual partners and frequency of testing. *African Health Sciences,**18*(3), 523–530. 10.4314/ahs.v18i3.8 PMC6307011 30602983

[CR30] Wagner, G., Bogart, L., Klein, D., Green, H., Kambugu, A., Nampiima, J., & Matovu, J. (2021). Examination of mediators and moderators to understand how and in what context Game Changers increases HIV prevention advocacy among persons living with HIV in Uganda. *International Journal of Behavioral Medicine,**28*(6), 737–745. 10.1007/s12529-021-09983-z PMC8481355 33791991

[CR31] Wagner, G. J., Bogart, L. M., Matovu, J. K. B., Gwokyalya, V., Green, H. D., Jr., Ninsiima, S., Klein, D. J., McBain, R. K., & Okoboi, S. (2025). HIV prevention through social networks: Evidence from a randomized controlled trial of a peer advocacy intervention in Kampala, Uganda. *Journal of HIV & Social Services*. 10.1080/15381501.2025.2598249 PMC12758887 41487462

[CR32] Wagner, G. J., Bogart, L. M., Matovu, J. K. B., Okoboi, S., Gwokyalya, V., Ninsiima, S., McBain, R. K., Storholm, E. D., Malika, N., & Green, H. D. (2024). Correlates of HIV prevention advocacy by persons living with HIV in Kampala, Uganda: A cross-sectional evaluation of a conceptual model. *International Journal of Behavioral Medicine*. 10.1007/s12529-024-10277-3 PMC11416567 38519810

[CR33] Webel, A. R. (2010). Testing a peer-based symptom management intervention for women living with HIV/AIDS. *AIDS Care,**22*(9), 1029–1040. 10.1080/09540120903214389 PMC3131156 20146111

